# Normal Body Temperature: A Systematic Review

**DOI:** 10.1093/ofid/ofz032

**Published:** 2019-04-09

**Authors:** Ivayla I Geneva, Brian Cuzzo, Tasaduq Fazili, Waleed Javaid

**Affiliations:** 1 State University of New York Upstate Medical University, Syracuse, NY, USA; 2 Department of Internal Medicine, Icahn School of Medicine at Mount Sinai, New York, NY; 3 Division of Infectious Diseases, Icahn School of Medicine at Mount Sinai, New York, NY; 4 Icahn School of Medicine at Mount Sinai, New York, NY

**Keywords:** body temperature, fever, hypothermia, normothermia

## Abstract

PubMed was searched from 1935 to December 2017 with a variety of search phrases among article titles. The references of the identified manuscripts were then manually searched. The inclusion criteria were as follows: (1) the paper presented data on measured normal body temperature of healthy human subjects ages 18 and older, (2) a prospective design was used, and (3) the paper was written in or translated into the English language. Thirty-six articles met the inclusion criteria. This comprised 9227 measurement sites from 7636 subjects. The calculated ranges (mean ± 2 standard deviations) were 36.32–37.76 (rectal), 35.76–37.52 (tympanic), 35.61–37.61 (urine), 35.73–37.41 (oral), and 35.01–36.93 (axillary). Older adults (age ≥60) had lower temperature than younger adults (age <60) by 0.23°C, on average. There was only insignificant gender difference. Compared with the currently established reference point for normothermia of 36.8°C, our means are slightly lower but the difference likely has no physiological importance. We conclude that the most important patient factors remain site of measurement and patient’s age.

Human body temperature is well established as one of the key vital signs. It is measured at regular intervals in the medical setting and often at home to try estimate the degree of “sickness” of an individual [1]. It had been used since antiquity [2–5], yet its interpretation had been, and still is, actively debated in the clinical setting [1, 6, 7]. The first step towards understanding the relationship between temperature and disease is to define “normal” body temperature, from where deviations can be measured. Indeed, many attempts had been made to this end, including the 1868 seminal paper by Wunderlich [8], who is believed to be the first to establish a link between fever and clinical diagnosis. He was also the first to apply a thermometer experimentally to measure human body temperature. Using a large sample size, Wunderlich [8] concluded that the average axillary temperature was 37.0°C, with the upper limit of normal defined as 38.0°C. However, newer studies challenged Wunderlich’s [8] “normothermia” [6]. Furthermore, research had shown that body temperature is a nonlinear function of several variables such as age, state of health, gender, environmental temperature, time of the diurnal cycle, among many others [9, 10]. To make the best use of the currently available literature, we reviewed and herein present an analysis of previously published human body temperature studies using healthy individuals, with the goal of better understanding the variables that determine normal body temperature.

## METHODS

The peer-reviewed literature was searched using PubMed (Table 1). The time period ranged from 1935 to December 2017. The following search phrases among article titles were used: “normal body temperature”, “body temperature AND review”, “body temperature AND adult”, “body temperature AND gender”, “human body temperature”, “core body temperature”, “hypothermia AND elderly”, “body temperature AND measurement”, “tympanic body temperature AND measurement”, “rectal body temperature AND measurement”, and “oral body temperature AND measurement”. Furthermore, the references of the above-identified papers were manually searched for additional useful articles. To be included in our analysis, papers had to meet the following inclusion criteria: (1) the paper presented data on measured normal body temperature of healthy human subjects ages 18 and older, (2) a prospective design was used, and (3) the paper was written in or translated into the English language. Using the data from the articles that met our inclusion criteria, we calculated mean temperatures and ranges before and after stratifying the data by gender, age (less than 60 years old vs 60 years old or older), and site of measurement (oral, axillary, temporal, rectal, urine) or by both variables.

**Table 1.** Summary of the Literature Data Search Grouped by Search Phrase

Search Phrase	No. of Articles Identified
normal body temperature	43
body temperature AND review	79
body temperature AND adult	47
body temperature AND gender	4
human body temperature	40
core body temperature	251
hypothermia AND elderly	108
body temperature AND measurement	110
tympanic temperature AND measurement	11
rectal temperature AND measurement	11
oral temperature AND measurement	10

Pooled standard deviations were calculated using the pooled standard deviation formula:

$$S_{\text{pooled}}=\sqrt{\frac{(n_{1}-1)S_{1}{}^{2}+(n_{2}-1)S_{2}{}^{2}+...+(n_{k}-1)S_{k}{}^{2}}{n_{1}+n_{2}+...+n_{k}-k}}.$$

For equal sample sizes, the formula was simplified as follows:

$$S_{\text{pooled}}=\sqrt{\frac{S_{1}{}^{2}+S_{2}{}^{2}+...+S_{k}{}^{2}}{k}}.$$

For the data in which standard deviation for the measured temperatures was not reported in the original articles, the standard deviation was estimated via extrapolation from a plot of the known standard deviations and the corresponding sample sizes. Table 2 shows the available and missing standard deviations (8 of the 36 articles that met our inclusion criteria did not report standard deviations for at least some portion of their data).

**Table 2.** Data Summary From the Articles That Met the Inclusion Criteria

Author	Study Year	Demographics	N	Measurement Site	Mean	Mean ± 2 SD
Baker [11]	1984	24 female students	24	Oral	36.8	36.058–37.542
Barley [12]	1970	Undescribed demographics	38	Oral	36.36	35.28–37.37
Basak [13]	2013	Healthy Asian student volunteers, mixed gender with an average age of 19.66	452	Oral	36.71	35.91–37.51
				Tympanic	36.78	36–37.56
Casa [14]	2007	Mixed gender, average age 26.5	25	Tympanic	37.16	36.585–37.725
Castle [15]	1993	NH residents (unknown gender) age 42–102	85	Oral	36.33	35.67–36.99
		NH residents (unknown gender) age 42–102	22	Rectal	37	36.222–37.778
Chamberlain [16]	1995	Age 16–65	1035	Tympanic	36.55	35.67–37.43
		Age 66–75	180	Tympanic	36.46	35.6–36.46
		Age 76–85	149	Tympanic	36.43	35.47–37.39
		Age >85	168	Tympanic	36.4	35.48–37.32
		All	1532	Tympanic	36.51	35.618–37.405
		All males	564	Tympanic	36.5	35.48–37.52
		All females	861	Tympanic	36.6	35.7–37.5
Collins [17]	1977	Age 69–90, measured during winter	47 (19 males, 28 females)	Oral	36.28	35.307–37.263
				Urine	36.51	35.69–37.334
Collins [18]	1981	Males, age 70–80	17	Oral	36.6	36–37.2
		Males, age 18–39	13	Oral	36.7	35.752–37.648
Doyle [19]	1992	Healthy healthcare worker volunteers, mixed gender	41	Rectal	37.7	36.9–38.5
				Oral	36.9	35.9–37.9
				Tympanic	36.1	34.9–37.3
Edwards [20]	1978	Healthy volunteers, mixed gender age 20–35	12	Tympanic	36.77	36.21–37.33
				Oral	37.1	36.54–37.66
				Rectal	37.36	36.8–37.92
Erickson [21]	1980	Hospital faculty between ages 18–42	50 (4 males, 46 females)	Oral	36.69	36.515–36.857
Erickson [22]	1985	Males age 57–75	760	Oral	36.73	35.89–37.57
Fox [23]	1971	Males age 12–28	12	Rectal	37.24	36.98–37.496
				Urine	37.09	36.624–37.548
				Oral	36.72	36.26–37.176
Fox [24]	1973	Mixed genders, age >65	1020	Oral	36.24	34.999–37.491
Fox [25]	1973	Mixed gender, age ≥65	72	Oral	36.1	34.9–37.3
				Urine	36.4	34.6–38.2
		Male only	20	Oral	36	34.8–37.2
				Urine	36.3	34.9–37.7
		Female only	52	Oral	36.2	35–37.4
				Urine	36.4	34.4–38.4
Gommolin [26]	2005	NH residents, mixed gender with an average age of 80.7	150	Oral	36.40	35.527–37.283
Gommolin [27]	2007	NH residents, mixed gender with an average age of 82.5	167	Oral	36.30	35.332–37.28
Gunes [28]	2008	NH residents, age 65–90	133	Axillary	35.77	34.5–36.5
Hasan [29]	2010	Mixed gender, average age 34	184	Axillary	36.39	35.61–37.5
				Oral	36.8	36.1–37.6
Higgins [30]	1983	Healthy volunteers, mixed gender age 65–90	60	Oral	36.61	
		Male only	27	Oral	36.72	
		Female only	33	Oral	36.61	
Horwath [31]	1950	Healthy male volunteers, age 16–37	16	Rectal	37.056	36.428–37.684
				Oral	36.53	35.978–37.078
		Healthy female volunteers, age 19–35	38	Rectal	37.14	36.747–37.531
				Oral	36.72	36.408–37.036
Ivy [32]	1945	Healthy medical students	276	Oral	36.7	35.8–37.4
Keilson [33]	1985	11 males, 9 females age 22–43	20	Urine	36.4	35.72–37.08
				Oral	36.21	35.41–37.01
		30 males, 65 females age 65–90	95	Urine	36.53	35.81–37.25
				Oral	36.41	35.57–37.25
Kolanowski [34]	1981	Mixed gender, age 65–97 reported in the winter	101	Rectal	36.66	34.4–37.6
				Oral	36.02	33.4–37.3
Linder [35]	1935	Male volunteers, medical staff, and researchers	24	Oral	36.64	36.564–36.708
				Rectal	37.14	37.044–37.244
Lu [36]	2009	Taiwanese volunteers, temperatures measured in winter and summer				
		Mixed gender, age 65–95	519	Oral	36.79	36.392–37.196
		Mixed gender, age 20–64	530	Oral	36.80	36.393–37.197
		Males, age ≥65	271	Oral	36.76	36.358–37.162
		Females, age ≥65	248	Oral	36.84	36.453–37.217
Mackowiack [6]	1992	Healthy volunteers, age 18–40	120	Oral	36.8	35.6–38.2
		Female	26	Oral	36.9	35.78–38.02
		Male	122	Oral	36.7	35.62–37.78
		African American	105	Oral	36.8	35.78–37.82
		White	43	Oral	36.7	35.48–37.92
Marion [37]	1991	Healthy volunteers, mixed gender age 64–96	93	Urine	37	36.5–37.5
				Oral	36.89	36.387–37.391
Marui [38]	2017	Mixed gender, Japanese volunteers with an average age of 20.7	141	Axillary	36.45	35.544–37.356
				Tympanic	36.8	36.2–37.4
McGann [39]	1993	Healthy African American females	35	Oral	36.94	36.42–37.46
		Healthy white females	41	Oral	36.81	36.39–37.23
		Healthy white males	16	Oral	36.79	36.37–37.21
Nakamura [40]	1997	Healthy Japanese nursing home residents, age ≥63	57	Oral	36.49	35.552–37.428
Salvosa [41]	1971	Women, age 69–93	40	Oral	36.02	34.81–37.23
Sund-Levander [42]	2002	Healthy volunters, mixed gender age ≥65	237	Rectal	37.05	35.6–38
				Tympanic	37.1	33.8–38.4
		Female only	159	Rectal	37.1	36.3–37.9
				Tympanic	37.15	36.046–38.254
		Male only	78	Rectal	37.05	36.342–37.758
				Tympanic	37	36–38
Terndrup [43]	1989	Healthy volunteers, mixed gender with an average age of 33.4	22	Oral	36.4	
				Rectal	37.1	36.9–37.3
				Tympanic	37.3	
				Tympanic	38.3	37.3–39.3
Thatcher [44]	1983	Mixed gender, age 60–94 measured in summer and winter	100	Oral	36.6	35.7–37.4
		Summer subset	50	Oral	36.8	36.3–37.4
		Winter subset	50	Oral	36.4	35.7–37
Thomas [45]	2004	Healthy females, age 21–36	19	Rectal	37.19	36.38–38
				Axillary	36.01	34.622–37.398
		Healthy females, age 39–59	74	Rectal	36.98	35.41–36.61
				Axillary	34.39	33.11–35.67

Abbreviations: N, number of participants; NH, New Hampshire; SD, standard deviation.

## RESULTS

**Figure 1.**
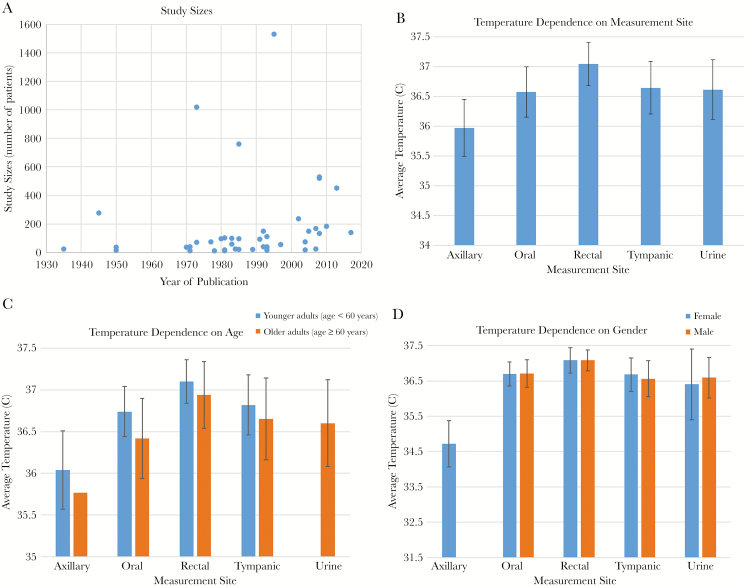
Literature search results and the determinants of normothermia. (A) Number of studies and their sizes over the search time period. (B) The dependence of body temperature on measurement site. (C) The dependence of body temperature on age, shown stratified by measurement site. (D) The dependence of body temperature on gender, shown stratified by measurement site.

The search hits are summarized in Table 1. A total of 36 articles met our inclusion criteria and the extracted raw data is shown in Table 2. The sample sizes for all of these studies were plotted against the year in which the studies were published in Figure 1A. Of the identified articles, 33 reported oral temperatures, 13 reported rectal temperatures, 9 reported tympanic temperatures, 6 reported urine temperatures, and 5 reported axillary temperatures. Seventeen of the studies reported temperatures in younger adults (age <60 years) and 19 reported temperatures in older adults (age ≥60 years). There were a total of 7636 healthy subjects, 1992 of which were identified as female and 2102 were identified as male, and the rest did not have their gender reported. There were a total of 9227 individual measurement sites used, where 5257 adults provided oral measurements, 2462 provided tympanic measurements, 618 provided rectal measurements, 551 provided axillary measurements, and 339 adults provided urine measurements. Our statistical analysis (Table 3) showed that the average body temperature among all subjects in all 36 studies and combining the data from all measurement sites was 36.59 ± 0.43 (standard deviation).

**Table 3.** Summary of Normal Body Temperature Ranges Stratified by the Modifying Factors Measurement Site, Age, and Gender

N	Number of Studies	Number of Individual Measurement Sites	Mean Temperature (°C)	Standard Deviation
All measurement sites, all subjects	36	9227	36.59	0.43
Stratification by Measurement Site				
Axillary	5	551	35.97	0.48
Oral	33	5257	36.57	0.42
Rectal	13	618	37.04	0.36
Tympanic	9	2462	36.64	0.44
Urine	6	339	36.61	0.5
Stratification by Age				
All measurement sites, all subjects <60 years	17	3114	36.69	0.34
All measurement sites, all subjects ≥60 years	19	4249	36.5	0.48
Stratification by Age and Measurement Site				
Axillary, subjects <60 years	4	418	36.04	0.47
Oral, subjects <60 years	15	1795	36.74	0.3
Rectal, subjects <60 years	8	217	37.1	0.26
Tympanic, subjects <60 years	5	652	36.82	0.36
Axillary, subjects ≥60 years	1	133	35.77	
Oral, subjects ≥60 years	18	2715	36.42	0.48
Rectal, subjects ≥60 years	3	360	36.94	0.4
Tympanic, subjects ≥60 years	4	734	36.65	0.49
Urine, subjects ≥60 years	4	307	36.6	0.52
Stratification by Gender				
All measurement sites, all female subjects	12	1992	36.65	0.46
All measurement sites, all male subjects	12	2102	36.69	0.43
Stratification by Gender and Measurement Site				
Axillary, female subjects	2	93	34.72	0.65
Oral, female subjects	9	537	36.7	0.34
Rectal, female subjects	4	290	37.08	0.36
Tympanic, female subjects	2	1020	36.68	0.47
Urine, female subjects	1	52	36.4	1
Oral, male subjects	11	1298	36.71	0.39
Rectal, male subjects	4	130	37.08	0.3
Tympanic, male subjects	2	642	36.56	0.51
Urine, male subjects	2	32	36.59	0.57

The average temperatures per measurement site, in decreasing order, were rectal at 37.04 ± 0.36, tympanic at 36.64 ± 0.44, urine at 36.61 ± 0.5, oral at 36.57 ± 0.42, and axillary at 35.97 ± 0.48 (Figure 1B, Table 3). Overall, when using the data from all of the measurement sites, the average body temperature of younger adults (<60 years of age) was higher (36.69 ± 0.34) than the average body temperature of older adults ( ≥60 years of age), which was 36.5 ± 0.48. The same age-related trend held true for all individual measurement sites (Figure 1C, Table 3). When looking at gender differences, we found that when using all reported measurements, the average body temperature of females was slightly lower (36.65 ± 0.46) compared with males (36.69 ± 0.43), but this trend was not pronounced when looking at the individual measurement sites, except for the urine measurement site (Figure 1D, Table 3).

## DISCUSSION

The quest for understanding human body temperature and defining normothermia is ongoing, as is evidenced by the steady number of published prospective studies depicted in Figure 1A. To the best of our knowledge, our systematic review, where we analyzed 36 separate prospective studies, is the largest of its kind. When using the data from all measurement sites and all included studies, we calculated the overall mean body temperature to be 36.59°C, which is lower than the currently acceptable mean of 36.8, as published in one of the most respected medical reference books, Harrison’s Principles of Internal Medicine [46]. However, the latter number from the reference book is not based on an all-inclusive meta-analysis, and therefore our average is likely more accurate. Of course, it should be kept in mind that there is no single number that defines normothermia; instead, there is a range for normal temperature, with corresponding standard deviation and standard error. As such, the 0.2°C difference in the mean when we compare our mean temperature with the Harrrison’s is likely not of much physiological relevance. In that respect, our calculated overall range (mean ± 2 standard deviations) is 36.16–37.02°C, which is narrower than the range of 33.2–38.3°C reported by Sund-Levander et al [42], which is an older systematic review comprising of only 20 studies, all of which were also part of our analysis. The tighter range is most likely due to bigger sample size used in our report, which validates our results further.

Knowing that body temperature is influenced by the measurement site, we calculated average temperatures, in decreasing order, rectal at 37.04°C, tympanic at 36.64°C, urine at 36.61°C, oral at 36.57°C, and axillary at 35.97°C. The trend is similar to the one reported by Sund-Levander et al [42]; however, the latter systematic review did not contain measurements of urine temperature. In addition, all of our site-specific calculated temperatures, except for axillary, were higher compared with the Sund-Levander et al [42] report. Furthermore, it is intriguing that we found such a large difference between what is considered the body core temperatures: rectal (37.04°C) and urine (urine at 36.61°C). This likely reflects a fault in the measurement in earlier studies from the 1970s and 1980s, which constitute a significant portion of the analyzed data and in which the measurements of urine temperature were not done invasively, eg, via a monotherm system. Therefore, these urine temperatures are fundamentally different from what we should consider core body temperature, which is temperature measured inside the human body.

With regards to age, our analysis confirmed that, on average, healthy elderly people have lower body temperature (Table 3 and Figure 1B) compared with younger adults. This was true for both the total average as well as for the individual measurements sites, except for urine temperatures because there were no studies reporting such measurements among younger adults. The decrease in body temperature with age is believed to be a phenomenon arising from a slowing of the human metabolic rate coupled with a decline in the ability to regulate body temperature in response to environmental changes such as seasonal changes, which had been previously studied [17, 19, 22, 47, 48]. These age-related changes are of particular clinical importance because elderly patients are often not capable of mounting a strong inflammatory response to infection and disease, with their temperature failing to reach the temperature range of what is traditionally considered the fever temperature range. Moreover, there is evidence to suggest that the presence of a robust fever response carries prognostic value when considering such infectious disease processes [49]. In the elderly, who may not be able to mount such a thermal response, we may similarly have to readjust our outlook on temperature-based prognostication. However, until we have research data to specifically address this question, clinicians should use lower normal temperature ranges as reference in the elderly, such as the ones presented in our systematic review.

Finally, our analysis demonstrated only a trivial difference in body temperature between the genders (Table 2 and Figure 1C), with women’s temperature being slightly lower when using all measurements from all measurement sites. However, when grouping the results by measurement site, in some cases (tympanic site) females’ body temperature is in fact higher compared with their male counterparts, whereas in other cases there is no difference (oral and rectal sites). There had been a disagreement in the literature as well, with some studies reporting that females have higher body temperature [6, 8, 16, 31], whereas others reported no differences among the genders [39]. Gender differences in body temperature had been suspected to relate to a difference in body fat percentage between women and men. Those studies revealed that women have a comparably larger percentage of body fat distribution subcutaneously, which in turn correlates with lower average skin temperatures [50, 51]. It had also been theorized that body temperature differences relate to female hormone levels, and yet, even in the studies that report statistically significant differences, the actual difference is fairly small and thus not likely to be of any clinical significance. Our large sample size from 36 individual studies is expected to reflect the true temperature variable in the human population and supports the lack of clinical significance of gender-based body temperature difference even if it could be measured.

## CONCLUSIONS

Human body temperature is a highly variable vital sign and known to be influenced by several variables, most prominently the person’s age and the site of measurement. Our systematic review is the largest of its kind and provides clinicians with evidence-based normal temperature ranges to guide their evaluation of patients with possible fever or hypothermia.
